# Community-based Study of Reproductive Tract Infections, Including Sexually Transmitted Infections, Among the Rural Population of Punjab, India

**DOI:** 10.4103/0970-0218.58401

**Published:** 2009-10

**Authors:** Neerja Jindal, Aruna Aggarwal, Paramjit Gill, Bableen Sabharwal, Babica Bhandari Sheevani

**Affiliations:** Department of Microbiology, Government Medical College, Amritsar, India

## Introduction

Reproductive tract infections (RTIs) including sexually transmitted infections (STIs) are recognized as public health problem and rank second as the cause of healthy life lost among women of reproductive age after maternal morbidity and mortality in developing countries.(1) Emerging epidemics of Acquired immunodeficiency syndrome (AIDS) and identification of STIs as a co-factor in its acquisition have made the control of these infections, one of the strategies imperative to decrease the transmission of HIV/AIDS. World over 340 million people are affected by STIs, out of which 30 million reside in India.(1) However baseline information on STI prevalence in India especially in general population which is necessary to formulate control strategies is lacking. Therefore the present community based study was carried out to determine the current prevalence rates of RTIs/STIs (HIV infection, genital herpes, syphilis, chlamydial infection, gonorrhea, trichomoniasis, candidiasis and bacterial vaginosis) in a rural setup.

## Materials and Methods

A total of 400 individuals (200 women and 200 men) in the age group of 15 – 45 years, who attended four different camps organized in four villages of Punjab, were screened for various RTIs / STIs.

After recording the relevant history, including the previous history of STI of that individual and the partner, a clinical examination was performed, and the following clinical specimens were collected after taking their consent:

1. Urethral swabs
2. Endocervical and vaginal swabs (in females). The presterilized swabs were used to collect the specimens
3. Urine samples were collected in a sterile universal container
4. Five milliliters of blood was collected aseptically, and the serum was separated and stored for further tests

To detect gonorrhea, urethral and endocervical swabs were subjected to

1. Gram staining (for gram negative intracellular diplococci)
2. Culture on chocolate agar and Thayer Martin medium.

Urine samples were transported to National Institute of Communicable Diseases (NICD), Delhi for polymerase chain reaction (PCR), for gonococci.

Vaginal secretions were tested for pH, sniffed for the presence of fishy odor, subjected to a whiff test and cultured on blood agar and MacConkey agar media for detection of bacterial vaginosis.

*Trichomonas vaginalis* was identified by its typical morphology and motility on the wet mount of vaginal secretion.

Vulvovaginal candidiasis was diagnosed by gram staining, potassium hydroxide (KOH) preparation, and culturing on Sabouraud dextrose agar (SDA).

For chlamydial infection, the urine samples were transported to NICD, Delhi, under the cold chain for the PCR amplification technique.

The serum samples were tested for (a) the presence of IgM antibodies to HSV-2 by the enzyme-linked immunosorbent assay (ELISA) test (Human kit provided by Wiesbaden, Germany), (b) Venereal Disease Research Laboratory (VDRL) slide flocculation test was done for syphilis, and (c) the ELISA test was performed for detecting HIV antibodies. The test was done according to the instructions given in the kit supplied by the National AIDS Control Organization (NACO) (Biotest, anti HIV, TETRAELISA). The samples reactive in the ELISA test were subjected to two other ELISA / rapid / simple tests, using different antigens. Sera reactive in three tests were taken as positive.

## Results

Out of the 400 individuals screened, 47 (11.75) were found to be positive for various RTIs. Of these 47, 40 (85%) were females and only seven (15%) were males. Table 1 shows that a majority of the positive individuals were more than 25 years of age (89.4%), illiterate, or had an educations less than primary level (87.3%), belonged to a low socioeconomic status (89.4%), and were married (93.6%). Of the 40 females, 31 (77.5%) were housewives and of the seven males, six (85.7%) were laborers by occupation. A history of having multiple sexual partners was given by five (71.42%) males, but not by any of the females. Past history of STIs (ulcer, wart, discharge, etc.) was present in only one (14.3%) male and eight (20%) females. In females, vaginal discharge and low back ache were very common presenting symptoms.

**Table 1:** Sociodemographic profile of 47 RTI / STI positive individuals

Charactristic	Chlamydia N=02	Gonorrhoea N=04	Bact. vaginosis N=22	Vulvo vaginal candiasis N=08	Genital herpes(Hsv-2) N=09	HIV N=02	Trichomonas	Syphilis	Total
Age (Years)									
<25	-	02	-	01	02	-	-	-	05 (10.6)
≥25	02	02	22	07	07	02	-	-	42 (89.4)
Sex									
Male	-	02	-	-	03	02	-	-	07 (15)
Female	02	02	22	08	06	-	-	-	40 (85)
Literacy									
≤Primary	-	03	22	08	07	01	-	-	41 (87.3)
>Primary	02	01	-	-	02	01	-	-	06 (12.7)
Marital status									
Married	02	03	21	08	08	02	-	-	44 (93.6)
Unmarried	-	01	01	-	01	-	-	-	03 (06.4)
Socio-economic status									
Low	02	03	20	07	08	02	-	-	42 (89.4)
Middle	-	01	02	01	01	-	-	-	05 (10.6)
Upper	-	-	-	-	-	-	-	-	00 (0.0)
Occupation									
House-wife	02	02	18	03	06	-	-	-	31
Labourer	-	01	04	05	03	02	-	-	15
Student	-	01	-	-	00	-	-	-	01
H/O MSF									
Self	-	02	-	-	01	02	-	-	05
Partner	-	-	-	-	-	-	-	-	-

N represents number of positive individuals, Figures in parenthesis represent percentage

In the present study, the prevalence rate of RTIs / STIs in females was 20% (40/200) and in males 3.5% (7/200) and no one was found to be suffering from more than one infection. The maximum positivity in females was that of bacterial vaginosis 11% (22/200) followed by candidiasis 4% (8/200), genital herpes (HSV-2 infection) 3% (6/200), and Chlamydia and gonorrhea 1% (2/200) each. No female was found to be suffering from HIV infection, syphilis or trichomoniasis. Both the HIV positive individuals were married males of low socioeconomic class, with a history of high-risk behavior. They were not aware of their HIV status and their spouses tested negative for HIV infection. Other STIs prevalent in the male population were HSV (1.5%) and gonorrhea (1%). Syphilis and trichomoniasis were altogether absent.

## Discussion

There are scattered reports regarding the prevalence of STIs in India, which vary in different population groups of geographically different regions of the country. In the present community-based study the observed prevalence rate was 11.75%, which is comparable to that from Tamil Nadu (14.6%).(2) Another STI prevalence study reported that the annual incidence rate of STIs in India was over 5%, and most regions of the country have relatively high levels of STIs.(1) Although these diseases cause suffering to both males and females, their prevalence is far more in females (20%) than in males (3.5%), in our study. Other studies have reported an even higher load of these infections in females.(3,4) This finding has very serious implications, because if left undiagnosed and untreated, the infections result in serious complications and sequelae, such as, infertility, fetal wastage, and malignancies in females. Their presence also compromises with contraceptive acceptance and continuation.

In the present study, the maximum number (89.40%) of STI positive individuals were above the age of 25 years [Table 1] and fell in the age group of 26 – 30 years. This is the time when a person is sexually very active and is also at the peak of his / her reproductive career. Hence, the damage caused by these infections is devastating, and is also supported by the study of Datey *et al*.(5) The finding that the majority (87.3%) of STI positive individuals of our study had a low level of education and belonged to the low socioeconomic status (89.4%), indicated that poverty and ignorance, along with the availability of few healthcare facilities in rural settings, played an important role in the high prevalence of STIs. The females were also constrained to seek medical assistance and faced several assess-related barriers. In a study conducted in Haryana village, 89% of the women who had symptoms of RTIs did not consult anyone for their treatment.(3) Similar to other studies, in our study too, high-risk behavior of the husband and previous history of STIs in self and husband [Table 1] were found to be associated with increased prevalence of STIs.(5) However, the risk factors alone could not accurately identify persons at risk of STIs within a community.

In females, vaginal discharge was the commonest symptom in our study. Eleven percent of these females had bacterial vaginosis and 4% vulvovaginal candidiasis. Other authors have also reported that these infections are important causes of vaginal discharge in our community.(3,4) However, we observed no case of Trichomonas infection, which was reported as 1.18% in the study of Rao *et al*.(4) The presence of genital herpes in 3% of the females in our study was important, because, apart from being a potential source of infection to others and to the child during birth, it was a risk factor for cervical neoplasia. Chlamydial infection and gonorrhea were observed in 1% of our female population, which was at variance with the findings of other authors.(3) The difference could be due to the fact that the techniques used were not similar. No female was found to be having gonoccocal infection, which corroborates the findings from other rural centers.(3,4) HIV infection was also not detected in females. This was similar to the findings from other areas of Punjab, where a very low rate of HIV infection was reported.(1)

In the present study, the prevalence of STIs in the male population was 3.5%, of which 1% had HIV infection. Migration, mobility, and highly promiscuous behavior, combined with ignorance were found to be responsible for a majority of the STIs in males. NACO reports that in India STIs combined with HIV infection in men, accounts for nearly 15% of healthy life lost.(1)

## Conclusion

In view of the above results it is concluded that we need to have cost-effective strategies for the early diagnosis and treatment of STIs and for their prevention, through information, education, and behavior change. These should form the basis of our strategies. This would go a long way in controlling the spread of HIV / AIDS and in reducing reproductive morbidity among the sexually active population of our country.
